# Supplementary material for: CAMDLES: CFD-DEM Simulation of Microbial Communities in Spaceflight and Artificial Microgravity
Source: Life (Basel). 2022 Apr 29;12(5):660. doi: 10.3390/life12050660 (PMC9144607; doi:10.3390/life12050660)
Supplement: Supplementary file 1 [file life-12-00660-s001.zip › life-1665717-supplementary/CAMDLES_supplement/CAMDLES_LIFE_ Supplemental_Tables.pdf]

life

Table S1: Physical, Chemical, and Biological Parameters. Default simulation parameters are used in all simulations unless otherwise stated.

Parameter	Symbol	Value	Units	Description	Ref.
**Simulation Physical Properties**					
Simulation Dimensions		300 x 100 x 300	µm	Radial and azimuthal periodic boundary conditions. Zero-gradient boundary conditions elsewhere	
Parcel Dimensions		5 x 20 x 5	µm	Fluid parcel size	
Simulation Volume	V	$9\ (10)^6$	μm^3		
Simulation Radius from HARV	r	$(10)^5$	µm	Maximum distance of simulation box to radius of rotation	
Fluid Density	ρ_f	1.00	$\frac{pg}{\mu m^3}$	Density of media	[54]
Fluid Kinematic Viscosity	ν	0.9	$\frac{\mu m^2}{\mu s}$	Constant viscosity of media	[54]
HARV Rotation Rate		10	R.P.M.		
Gravitational Field Strength	g	$9.81\ (10)^{-6}$	$\frac{pg}{\mu s^2}$		
**Integration Timesteps:**			µs		
Particle Timestep	dt	150-250	µs		
Fluid Timestep	dt	150-250	µs		
Chemical Timestep	dt	150-250	µs	Higher diffusion requires a smaller timestep	
Biological Timestep	dt	150-250	µs		
Temperature	T	310.15	K	Constant	[20]

**Simulation Chemical Properties**					
Mass Transfer Coefficient	h_s	variable	$\frac{\mu m}{\mu s}$		
Methionine Diffusivity	$D_{e,m}$	Low: 3.1 (10)$^{-4}$<br>Mid: 5.0 (10)$^{-4}$<br>High:7.6 (10)$^{-4}$	$\frac{\mu m^2}{\mu s}$	Fick's Law diffusion constant	[51, 55]
Acetate Diffusivity	$D_{e,a}$	Low: 4.9 (10)$^{-4}$<br>Mid: 5.0 (10)$^{-4}$<br>High:12. (10)$^{-4}$<br>4	$\frac{\mu m^2}{\mu s}$	Fick's Law diffusion constant	[51, 55]
Bulk Media Diffusivity	$D_{e,l}$	Low: 2.1 (10)$^{-4}$<br>Mid: 5.0 (10)$^{-4}$<br>High:5.1 (10)$^{-4}$	$\frac{\mu m^2}{\mu s}$	Fick's Law diffusion constant	[56]
Starting Methionine Quantity		0.00001/Y_{ES}	$\frac{pg}{\mu m^3}$	To kickstart metabolism, total Simulation Starting Quantity Distributed Equally among all Cells, in terms of Biomass Equivalents of *E*.	
Starting Acetate Quantity		0.00001/Y_{SS}	$\frac{pg}{\mu m^3}$	Starting Quantity in terms of Biomass Equivalents of *S*.	
Acetate Coefficient of Solutal Specific Gravity	β_1	0.85		Linearly interpolated coefficient that converts acetate concentration (cell equilvalent) to fluid density	[57]
Methionine Coefficient of Solutal Specific Gravity	β_2	0.003		Linearly interpolated coefficient that converts methionine concentration (cell equilvalent) to fluid density	[58]
Bulk Media Coefficient of Solutal Specific Gravity	β_3	0.39		Linearly interpolated coefficient that converts lactose concentration (cell equilvalent) to fluid density	[59]
**Bacterial Cell Physical/Mechanical Properties**					
Cell size	d	1.12	pg	Diameter, based on effective volume of a rod shaped *E. coli*	[60]

Cell Volumetric density	ρ_s	1.08	$\frac{pg}{\mu m^3}$	Needed for Bouyancy	[60]
Young's Modulus of Elasticity	E	50,000	$\frac{pg}{\mu m \mu s^2}$	Required LIGGGHTS® parameter	[46]
Coefficient of Restitution	e	0.05		Minimum Required LIGGGHTS® collision parameter	
Poisson Ratio	ν	0.4		Required LIGGGHTS® parameter	[46]
Surface Energy Intensity	γ	0	$\frac{pg}{\mu s^2}$	Cell – cell Adhesion Energy	
Coefficient of Friction	μ_f	0		Cell – cell Sliding Friction Coefficient	
			***E. coli* Biological Properties**		
Population Density		$1.11 (10)^{-10}$	$\frac{cells}{\mu m^3}$	Starting Density of Coculture	[20]
Maximal growth-rate	$\mu_{max,E}$	$1.82 (10)^{-11}$	μs^{-1}	Monod Kinetic Maximal Growth Rate	[29, 61]
Biomass Yield (Methionine)	Y_{ES}	70.2	$\frac{pg}{pg}$	Biomass/Methionine Mass	[17, 48]
Biomass Yield (Lactose)	Y_{ES}	1.5	$\frac{pg}{pg}$	Biomass/Lactose Mass	[61, 62]
Product Yield	Y_{PS}	31.9	$\frac{pg}{pg}$	Acetate/Methionine Mass	[29, 63]
Half-saturation constant	K_S	variable	$\frac{pg}{\mu m^3}$	Monod Kinetic Substrate Concentration of Half-maximal Growth Rate	[48]
			***S. enterica* Biological Properties**		
Population Density		$1.11 (10)^{-10}$	$\frac{cells}{\mu m^3}$	Starting Density of Coculture	[20]
Maximal growth-rate	$\mu_{max,S}$	$9.09 (10)^{-12}$	μs^{-1}	Monod Kinetic Maximal Growth Rate	[61]
Biomass Yield	Y_{SS}		$\frac{pg}{pg}$	Biomass/Acetate Mass	[61]

		0.46			
Product Yield	Y_{PS}	0.058	$\frac{pg}{pg}$	Methionine/Acetate Mass	[29, 61]
Half-saturation constant	K_S	variable	$\frac{pg}{\mu m^3}$	Monod Kinetic Substrate Concentration of Half Maximal Growth Rate	[48]
**Non-dimensional Numbers**					
Reynolds Number	Re	$\frac{u_f d}{\nu}$		Ratio of Inertial to Viscous Forces	
Schmidt Number	Sc	$\frac{\nu}{D_m}$		Ratio of Momentum Diffusivity to Mass Diffusivity	
Sherwood Number	Sh	$\frac{h}{D_m/d}$		Ratio of Convective to Diffusive Mass Transport	

Table S2: Simulation Details and Results. Simulation input files can be found in File S1.

File Names	Spatial Structure & Diffusivity	Population Size & E:S Ratio	Notes	Steady % Growth Rate
3.1.1a-c	Manually Placed,<br>Intermediate Diffusion	2 cells, 1 E : 1 S	0.01_starting metabolites<br>a. (1 μm distance)<br>b. (50 μm distance)<br>c. (218 μm distance)	4.7<br>4.6<br>4.4
3.1.1d,<br>3.1.1e/3.1.2a/3.1.3f	Manually Placed,<br>High Diffusion	3 cells, 2:1	0.00001_starting metabolites<br>d. (207 μm distance)<br>e. (1 μm distance)	0.8<br>4.9
3.1.2b-c,	Randomly Distributed<br>High Diffusion	b. 2000 cells 1.86:1<br>c. 283 cells 1.86:1		27.2<br>29.4
3.1.2c2,<br>3.1.2e	Spherical gaussian distributed<br>High Diffusion	C2. 283 cells 1.86:1<br>E. 100 cells 1.86:1		27.8<br>24.5
3.1.2d/3.1.3a/3.2.3e<br>3.1.3b-e	Tightly packed<br>Sphere, High diffusion	1:86:1<br>a. 2000 cells<br>b. 1000 cells<br>c. 500 cells<br>d. 283 cells<br>e. 99 cells<br>f. See above		35.4<br>29.3<br>28.8<br>26<br>23.4

3.1.3g/3.2.3c<br>3.1.3h-l	Tightly packed Sphere, Low diffusion	1:86:1<br>g. 2000 cells<br>h. 1000 cells<br>i. 500 cells<br>j. 283 cells<br>k. 99 cells<br>l. 3 cells		29.4<br>29.0<br>28.6<br>27.8<br>24.5<br>6.6
3.1.4a/3.2.3a<br>3.1.4b	Intermediate diffusion<br>a. Gaussian Distributed Sphere<br>b. Randomly distributed	2000 cells, 44 E : 1 S	0.00000001 starting metabolites<br>190% λ_E and 333% λ_S<br>Measured S. Growth	50<br>~0
3.2.1/3.2.3b			Same conditions as 3.1.4a plus RWV hydrodynamics	7.6
3.2.2a-b	Gaussian Distributed Spheres<br>Intermediate diffusion	2000 cells, 18 E : 1 S	0.00001 starting metabolites<br>190% λ_E and 333% λ_S<br>Measured S. Growth (218 µm distance)<br>a. Microgravity<br>b. RWV	not steady (Figure 8b)<br>not steady (Figure 8b)

3.2.3c-g	Tightly packed spheres,<br>c-e. Low Diffusion<br>f-g. High Diffusion	2000 cells,<br>1.86 E : 1 S	b. RWV Liquid colony<br>c. RWV Solid colony<br>d. Microgravity<br>e. RWV Solid Colony<br>f. Microgravity<br>λ_E and λ_S varies	Growth depends on product yield (Figure 9)
3.3.1a-b	Intermediate Diffusion<br>a. Flat cylindrical on floor<br>b. Spherical on floor	2000 cells,<br>44 E : 1 S	1g Gravity	Not steady state (Figure 8b)

6
